# Research on talent cultivation for rural revitalization based on three-party evolutionary game

**DOI:** 10.1371/journal.pone.0313827

**Published:** 2024-11-26

**Authors:** Jinxia Wang, Yunfeng Tan, Qiong Hu, Huarong Cheng, Fang Gao

**Affiliations:** 1 College of Resources and Safety, Chongqing Vocational Institute of Engineering, Chongqing, China; 2 College of River and Ocean Engineering, Chongqing Jiaotong University, Chongqing, China; 3 Chongqing Banan Yucai Experimental Secondary School, Chongqing, China; 4 Infrastructure Logistics Office, Chongqing Vocational Institute of Engineering, Chongqing, China; Zhejiang Shuren University, CHINA

## Abstract

The sustainable development of Rural Revitalization Talent Training (RRTT) is a key prerequisite for realizing the rural revitalization strategy. In order to study the influence of various stakeholders on RRTT, explore its optimal development path, and clarify the key control factors, this study analyzes the behavioral decision-making of RRTT stakeholders for the first time, and constructs an evolutionary game model of the government, universities and village collectives. Through dynamic decision replication analysis and evolutionary stability analysis, the game relationship among the three stakeholders is discussed. Furthermore, through numerical simulation, the decision-making characteristics of the three parties "behaviors and the evolution trend of the stakeholders" behaviors under the current situation are evaluated, the sensitivity of the key control factors with the policy changes is analyzed, and the feasibility of its implementation is discussed. The results show that the input cost of village collectives is the main determinant of RRTT, and appropriate financial input and low incentive policies are more conducive to universities and village collectives to actively promote RRTT. The research results provide decision-making basis for the implementation of rural revitalization strategy.

## 1 Introduction

**Fig 1:**
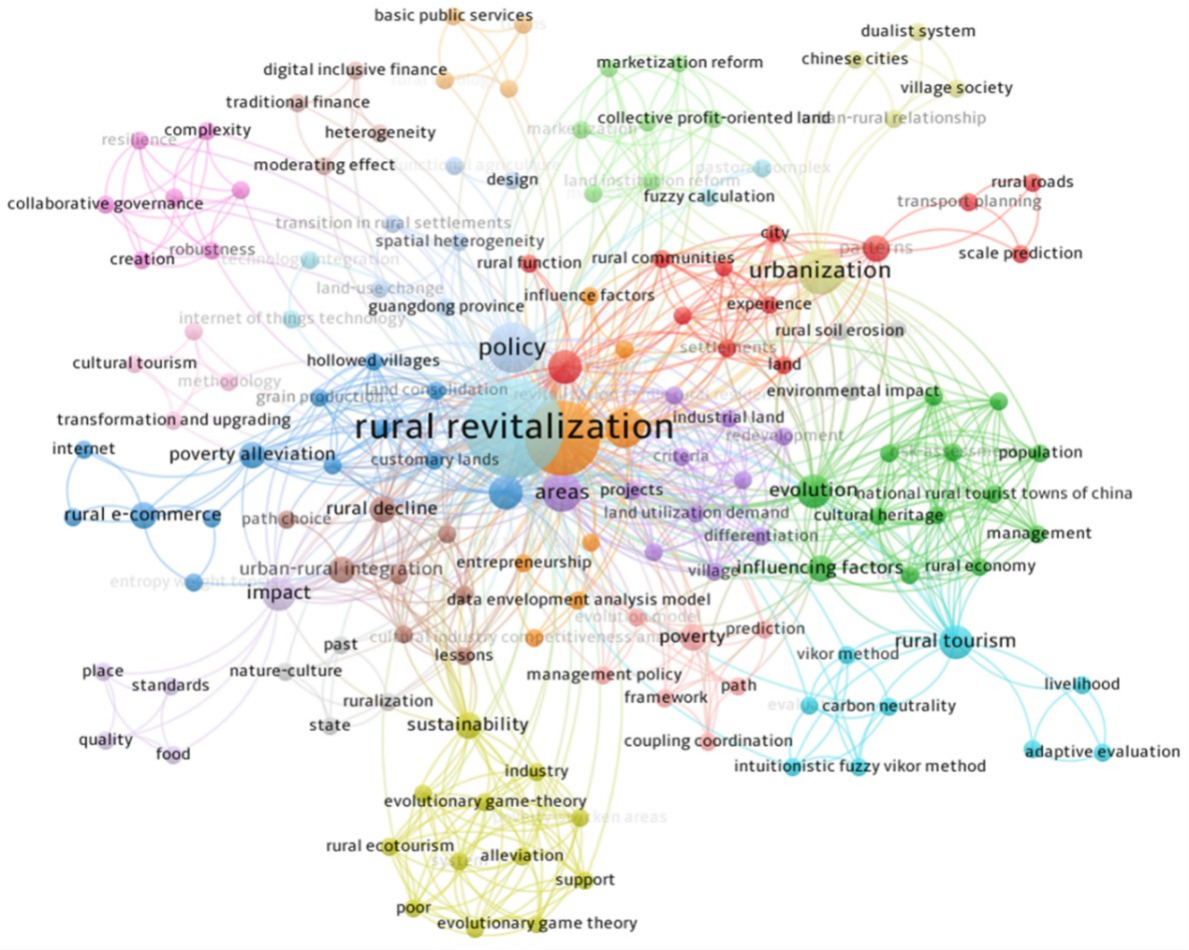
Statistical chart of academic research results of scientometrics.

Under the background of accelerating global urbanization, rural revitalization has become an important measure for countries to achieve coordinated regional development and agricultural and rural modernization [1]. According to statistics, more than 60 countries around the world have formulated and implemented rural revitalization strategies [2]. As the world’s largest developing country, China attaches great importance to rural revitalization, and in 2017, it rose to a national strategy, systematically deployed and comprehensively promoted [3]. Rural revitalization is not only an economic development issue, but also attracts the attention of many scholars and research institutions, and has become a research hotspot in academia [4]. Through the quantitative analysis of the Web of Science database, it is found that the existing research mainly focuses on national policies, rural economy, ecological environment, agricultural production and development path (as shown in Fig 1, while the systematic discussion of Rural Revitalization Talent Training (RRTT) is still insufficient.

As we all know. Strengthening the village with talents is the key to implementing the strategy of rural revitalization, and it is also the fundamental way to solve the "rural disease" and stimulate the new kinetic energy of rural development [5, 6]. However, the current RRTT faces many difficulties: the reason is that the government-led top-down management model is difficult to adapt to the diverse needs of rural development [7]. At the same time, the government, universities and village collectives have asymmetric information, different interest demands, and lack of effective communication and coordination mechanisms [8]. In addition, the enthusiasm of rural participation is not high, the willingness to cooperate is not strong, and the situation of co-construction and sharing has not yet formed [9]. In the end, the university RRTT is out of line with the actual needs of the countryside, and the contradiction between supply and demand is prominent. Therefore, in the process of RRTT, how to avoid the above difficulties and create a cooperative mechanism of government-led, benign interaction between universities and village collectives is an important measure to achieve rural revitalization.

In this context, this study aims at the sustainable development of RRTT, uses the evolutionary game model to identify key influencing factors, and quantifies the responsiveness of each subject’s strategy changes to parameters, and then simulates the changes in system stability. As a policy simulation study, this study not only helps to reveal the impact mechanism of multi-party interaction on the promotion of RRTT, but also accelerates the promotion of RRTT and provides useful enlightenment for future policy simulation research and suggestions.

The structure of this paper is as follows: Section 2 demonstrates the contribution of this study on the basis of reviewing the relevant literature. Section 3 elaborates on the method. The simulation results are demonstrated and analyzed in Section 4. In order to better illustrate the novelty and policy implications of this study, it is discussed in section 5 and summarized in section 6.

## 2 Literature review

### 2.1 Confusions in the development of RRTT

Universities play an indispensable role in RRTT [10]. Although universities have invested a lot of resources in theoretical teaching and personnel training, the existing education model still has a tendency to emphasize theory and despise practice [11]. This unbalanced teaching method leads to the relative lack of practical ability and skills of graduates, which is difficult to fully adapt to the work needs of rural agriculture [12].

Most of the universities are located in cities and far away from rural communities [13], which virtually limits the interaction between students and rural areas during their study, so that there is a certain deviation or lack of understanding of the actual needs and challenges in rural areas [14, 15]. College graduates tend to choose urban employment opportunities and have low enthusiasm for rural agricultural work [16, 17]. This is in contradiction with the talent demand of rural revitalization, which leads to the increasingly serious problem of brain drain or unreasonable distribution of talents. The unreasonable employment mechanism and concept also hinder the effective development of RRTT [18].

In addition, the university training mechanism is not perfect, the problem of rural brain drain is serious, the lack of rural education resources and other issues, the existence of these problems also seriously restrict the quality of RRTT. It is difficult to achieve the healthy development of RRTT by relying solely on the efforts of the university as a single subject, and multi-party coordination and systematic solutions are urgently needed.

### 2.2 The RRTT process involves the balance of interests of multiple subjects

Although the government has issued guidance on RRTT, there is still a lack of mature and comprehensive system framework and unified standards [19]. In addition, incentive policies, such as subsidies, incentives and tax breaks, are considered particularly important [20, 21]. However, since government investment and incentive policies will bring economic burden to the government, it is necessary to limit its coverage and intensity [22].

The information asymmetry among the government, universities and village collectives, the different interest demands, and the lack of effective communication and coordination mechanisms are the main reasons that affect RRTT. Rural areas need more talents with practical skills [5, 23], rather than relying solely on experts and scholars or short-term lectures [24, 25]. The village collective hopes that the government can provide high subsidies to support talents to the countryside [26], while worrying that the cost of external technicians is too high [27, 28].

In addition, the continuity of technology implementation has also received attention [29, 30]. For example, the application of new technologies may require the liberalization of land use, which involves complex rural collective land rights and interests [31, 32]. In addition, the enthusiasm of rural subjects to participate in RRTT is not high, their willingness to cooperate is not strong, and they have not formed a platform for resource sharing with universities. Therefore, the government strategy should also consider how to motivate talents to go to the countryside and ensure the continuous implementation of technology. Therefore, RRTT is a typical complex system involving multiple stakeholders (such as government, universities, village collectives, etc.). It is urgent to explore scientific and effective research methods that can balance the behavior of each subject, so as to provide RRTT strategies and practical paths for the government.

### 2.3 Research progress of evolutionary game theory in RRTT

The evolutionary game method plays an important role in studying and coordinating the complex relationship between multiple game subjects, especially in simulating the risk strategies and coping behaviors of different participants [33]. Compared with static games, dynamic games can provide a dynamic framework for behavioral strategy analysis [34]. In recent years, evolutionary game theory has attracted much attention in the field of talent training. The training mode of leisure sports talents is inspired by evolutionary game theory. By formulating classification standards, it provides a scientific basis for the training of rural sports professionals [35]. In addition, in the process of studying the coordinated development of government, enterprises and universities, the application of game evolution model improves the efficiency of tripartite cooperation and broadens their cooperation channels [36]. However, the dynamic evolutionary game method is affected by many factors when studying the multi-agent game problem of government participation [37]. For example, the government adjusts the system balance through positive strategies such as financial support, incentive subsidies and policy incentives [38], supplemented by coercive interventions such as punishment, thereby improving the overall decision-making efficiency of the government. However, various government strategies have different costs. Therefore, in the game process, it is particularly critical to clarify the impact of government strategies on the participants of RRTT [39]. In addition, the responsiveness and sensitivity of different subjects to changes in policy and external environment are quite different [40, 41]. Therefore, in the process of constructing the RRTT game model, the identification of influencing factors, parameter setting and application scenarios are the main factors that need to be seriously considered [42]. Therefore, the application of the game model can effectively identify the main influencing factors and provide a reference for the government’s relevant decision-making [43].

### 2.4 The shortcomings of RRTT research and the significance of this study

Scholars have explored and analyzed the evolution process, influencing factors and game relationship among RRTT subjects, which provides a rich theoretical basis for this paper. However, there are still some deficiencies in the existing research. On the one hand, it mainly focuses on the interest game between the talent demand subject and the university and between the government and the talent demand subject [44], while ignoring the balance role of the government in the tripartite game process. On the other hand, the main factors affecting decision-making efficiency are not fully discussed [36], resulting in unclear interest needs, asymmetric decision-making information and unequal income distribution [45, 46]. In addition, insufficient consideration is given to the cost differences of different government strategies and the differences in the response and sensitivity of each subject to government support.

Our research contributions are mainly reflected in two aspects. First of all, we propose a variability-payment coefficient of government funding related to RRTT to expand the strategic dimension of government funding. Secondly, according to the attributes of the university strategy, the input variable factors are introduced into the model when the university is affected by the government’s reward and punishment measures, and set as variables. Specifically, the payment coefficient of university investment refers to the response of willingness to pay caused by the change of strategy caused by government investment, reward and punishment factors. The newly proposed variables contribute to the model construction of similar topics.

## 3 Evolutionary game modeling

### 3.1 Model assumption

Reviewing the previous literature, most scholars only studied the development of talent training from a certain perspective, and did not integrate the mutual influence of multiple subjects involved in RRTT. Chen [47]conducted a correlation analysis on the level of talent training and its influencing factors, and proposed a new talent training model to provide a theoretical basis for the sustainable development of rural tourism. Some studies have also pointed out the importance of government intervention for the sustainable development of rural revitalization and talent development. As a national authority, the government can reasonably plan the layout of rural development and promote the sustainable development of rural economy [48]. The decentralized management of local governments will also have a great impact on rural development [49]. It is important that universities are the main places for personnel training and an important force for rural revitalization personnel training [50]. In addition, the talent training work is more inseparable from the active participation of the village collective. The village collective can provide resource support, practical opportunities and demand feedback for talent training, so as to promote the connection between the training of rural revitalization talents and the actual needs [51]. The cooperation and interaction of these three parties can promote the smooth progress of talent training in rural revitalization and promote the implementation of rural revitalization strategy. The difference, however, is that the government, universities and village collectives are generalized concepts and the three parties involve many participants, implementers and managers. For example, the government consists of local governments at all levels, covering education, supervisory and regulatory authorities, such as the Department of Education, the Bureau of Education, etc [52]. Universities include different levels of institutions of higher learning, including undergraduate colleges, colleges and vocational education institutions, as well as scientific research institutions [53, 54]. The village collective covers all administrative villages with different regions, economic levels and customs [55, 56]. The above specific participants all have mutual connections and games. Therefore, in order to unify their behavior and decision-making, we select the organizations (government, universities and village collectives) that best represent their interests and aspirations from the specific participants of each party as representatives in the study. In order to further explore and clarify the interaction mechanism among the government, university and village collective, the three-party evolutionary game model is introduced into RRTT development research. A framework system for the evolutionary relationship between government, university and village collective behavior in RRTT development is constructed.

**Fig 2:**
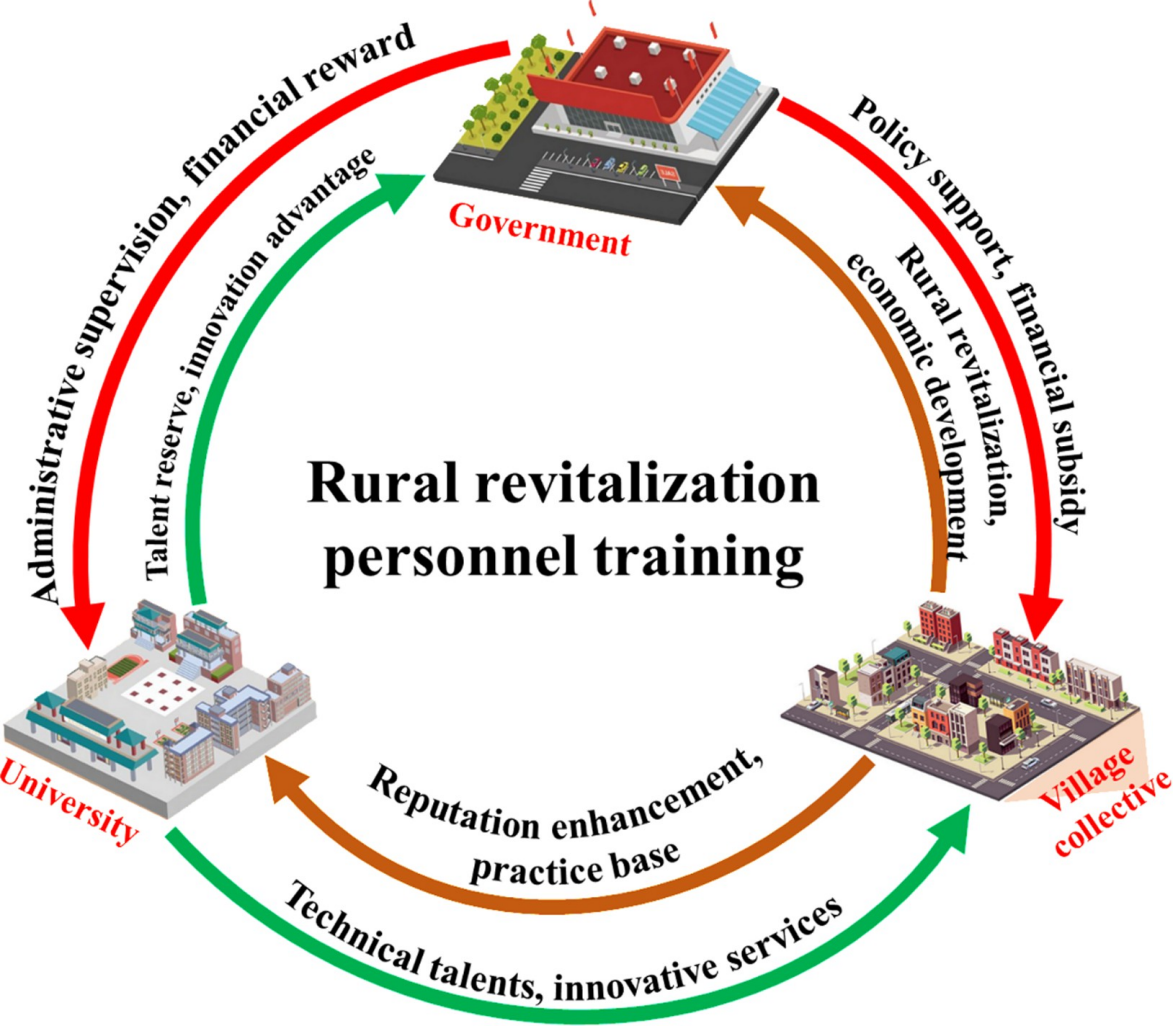
Stakeholder game framework for RRTT.

However, before constructing a three-way evolutionary game model, it is necessary to identify the stakeholders and the rules of the game in the system. The relationships between the three stakeholders and the rules of the game are described in the RRTT system (Fig 2). Regarding the sustainable development of China’s RRTT, the government, universities and village collectives are key stakeholders. In the role of the three, the village collective provides a practical base for the cultivation of talents in universities, but at the same time, it also hopes that universities will provide them with technical talents and innovative technologies. And obtain certain policy support and financial subsidies from the government, so as to achieve innovative development capabilities and unique development advantages. In order to maximize the economic benefits and social influence. Universities obtain financial support, improve the quality of personnel training, transform technology and innovation achievements, improve social service ability and enhance school reputation. At the same time, it provides the government with a talent pool for rural revitalization and forms innovative advantages. Through policy guidance, incentives, subsidies, supervision and other measures, the government enhances the motivation of universities and village collectives, and ultimately improves the quality and quantity of talent training. It reserves talents for rural revitalization, promotes the innovative development of villages and strengthens the regional economy.

To summarize, the government, the university and the village collectives are the three key stakeholders in the system, and the strategy of any one of the subjects affects the strategies of the other two. A three-party evolutionary game model is constructed below based on their strategies.

Hypothesis 1: The game in this paper involves three participating objects: the government, universities and village collectives, assuming that each party has two game strategies to choose from. That is, the government can choose "strong dominant" or "weak dominant" strategy. Universities can choose "active implementation" or "negative implementation" strategy. The village collective can choose the strategy of "efficient implementation" or "inefficient implementation". The government’s "strong dominant" strategy is defined as the local government’s strong support and investment in RRTT (*α*_1_), which mainly consists of monitoring the implementation of universities. Universities are rewarded for "active implementation" and penalized for "negative implementation". Rewards for "efficient implementation" and penalties for "inefficient implementation" by village collective. To promote the smooth implementation of RRTT. At the same time, the government gains the benefits of economic development. The government’s "weak dominance" strategy is defined as the local government’s support for RRTT, but with limited input (*α*_2_), when the government no longer rewards universities for "active implementation" and village collectives for "efficient implementation". The government no longer rewards universities for "active implementation" and village collectives for "efficient implementation", but still regulates the implementation of universities and village collectives. Universities are still penalized for "negative implementation" and "inefficient implementation".
Hypothesis 2: From the government’s perspective, when the university chooses to implement the "active implementation" strategy and the village chooses to implement the "efficient implementation" strategy, the government’s benefit is greater when the RRTT is running smoothly than when the RRTT is not running smoothly.
Hypothesis 3: From the perspective of universities, when village collectives choose the strategy of "efficient implementation", universities can choose the benefits of "active implementation" over the benefits of "negative implementation"; when village collectives choose the strategy of "inefficient implementation", universities can choose the benefits of "negative implementation" over the benefits of "active implementation".
Hypothesis 4: From the perspective of the village community, when the university chooses an "active implementation" strategy, the village community’s benefits from "efficient implementation" outweigh the benefits from "inefficient implementation"; when the university chooses a "negative implementation" strategy, the village community’s benefits from "inefficient implementation" outweigh the benefits from "efficient implementation".

Based on the above assumptions, all the variables used in the model and their meanings are shown in Table 1.

**Table 1:** Variables and meanings related to the three-way game.

Variable symbol	Meaning of variable
X	The probability of strong government dominance. 0≤x≤1
Y	The probability of active implementation in the universities. 0≤y≤1
Z	The probability of efficient implementation of village collectives. 0≤z≤1
α	The coefficient of payment of G_1_. strong government dominance (*α*_1_), weak government dominance (*α*_2_).0≤*α*_i_≤1, *α*_1_>*α*_1_
β	The coefficient of payment of E_1_. university positive (*β*_1_), university negative (*β*_2_). 0≤*β*_*i*_≤1, *β*_1_>*β*_2_
G_1_	Special funds for government RRTT training.
G_2_	The government gives village collective incentive subsidies.
G_3_	The government gives incentive subsidies to universities.
F_1_	The government’s punishment for the negative implementation of the universities.
F_2_	The government’s punishment for inefficient implementation of village collectives.
B	Gains to the government
E	Benefits obtained by universities
E_1_	Investment in universities
E_2_	When the university is positive and the village collective is inefficient, the negative benefits obtained by the university are negative.
E_3_	When the university is negative and the village collective is efficient, the negative benefits obtained by the university are negative.
V	When all three are negative, the negative effects of the village collective extra gain.
V_1_	Universities are negative, village collectives are efficient, and village collectives have negative effects.
V_2_	Universities are active, village collectives are inefficient, and village collectives have negative effects.
u	The government is strongly dominated, the village collective is efficient, and the village collective gives the cost of universities.

### 3.2 Model construction

Based on the above assumptions, eight groups of game combination strategies are selected for Government, Universities and Village collective game strategies. The specific income analysis is shown in Table 2.

**Table 2:** Combination of strategies and payoffs of the three-way game.

Strategy combination	Government (x)	Universities (y)	Village collective (z)
(Strong dominant, Active implementation, Efficient implementation)	B-*α*_1_G_1_-G_2_-G_3_	E+G_3_+V_3_ -*β*_1_E_1_	G_2_-V_3_
(Strong dominant, Active implementation, Inefficient execution)	B+F2-*α*_1_G_1_-G_3_	E+G_3_ -*β*_1_E_1_-E_2_	-V_2_ -F_2_
(Strong dominant, Negative implementation, Efficient implementation)	B+F_1_-*α*_1_G_1_-G_2_	E+V_3_ -*β*_1_E_1_ -E_3_-F_1_	G_2_-V_1_-V_3_
(Strong dominant, Negative implementation, Inefficient execution)	F_1_+F_2_-*α*_1_G_1_	E -*β*_1_E_1_-E_2_-E_3_-F_1_	-V_1_-V_2_ -F_2_
(Weak dominant, Active implementation, Efficient implementation)	B -*α*_2_G_1_	E-*β*_1_E_1_	0
(Weak dominant, Active implementation, Inefficient execution)	B -*α*_2_G_1_+F_2_	E-*β*_2_E_1_-E_2_	-V_2_ -F_2_
(Weak dominant, Negative implementation, Efficient implementation)	B -*α*_2_G_1_+F_1_	E-*β*_2_E_1_-E_3_-F_1_	-V_1_
(Weak dominant, Negative implementation, Inefficient execution)	F_1_+F_2_-*α*_2_G_1_	E-*β*_2_E_1_-E_2_-E_3_-F_1_	-V-V_1_-V_2_ -F_2_

## 4 Model replication dynamics and evolutionary stability analysis

### 4.1 Replicative dynamic modeling of game subjects

Since the government, the university and the village collective are all finite rational, they all want to maximize their own interests, analyze the dynamic adjustment of the game subjects and each subject’s strategy, construct a replicated dynamic model of each game subject, and analyze the evolutionary stabilization strategy.

Let the expected rate of return for the government’s "strong dominant" strategy be *U*_*g*1_, and the expected rate of return for the " weak dominant " strategy be *U*_*g*2_, with an average expected return of *U*_*g*_, as follows:

$$\begin{array}{l} U_{g1}=yz\left(B-\alpha_{1}G_{1}-G_{2}-G_{3}\right)+y(1-z)\left(B+F_{2}-\alpha_{1}G_{1}-G_{3}\right)\\ +(1-y)z\left(B+F_{1}-\alpha_{1}G_{1}-G_{2}\right)+(1-y)(1-z)\left(F_{1}+F_{2}-\alpha_{1}G_{1}\right) \end{array}$$

$$\begin{array}{l} U_{g2}=yz\left(B-\alpha_{2}G_{1}\right)+y(1-z)\left(B-\alpha_{2}G_{1}+F_{2}\right)+(1-y)z\left(B-\alpha_{2}G_{1}+F_{1}\right)\\ +(1-y)(1-z)\left(F_{1}+F_{2}-\alpha_{2}G_{1}\right) \end{array}$$

$$Ug=xU_{g1}+(1-x)U_{g2}$$

Therefore, the replication dynamic equation for the government’s "strong dominant" strategy is:

$$F(x)=\frac{dx}{dt}=x\left(U_{g1}-U_{g2}\right)$$

Similarly, the expected rate of return for universities choosing the "active implementation" strategy is *U*_*c*1_, while the expected rate of return for universities choosing the "negative implementation" strategy is *U*_*c*2_, and the average expected return is *U*_*c*_, as follows:

$$\begin{array}{l} U_{c1}=xz\left(E+G_{3}+V_{3}-\beta_{1}E_{1}\right)+x(1-z)\left(E+G_{3}-\beta_{1}E_{1}+E_{2}\right)\\ +(1-x)z\left(E+V_{3}-\beta_{1}E_{1}+E_{3}-F_{1}\right)+(1-x)(1-z)\left(E-\beta_{1}E_{1}-E_{2}-E_{3}\right.-F_{1}) \end{array}$$

$$\begin{array}{l} U_{c2}=xz\left(E-\beta_{2}E_{1}\right)+x(1-z)\left(E-\beta_{2}E_{1}-E_{2}\right)+(1-x)z\left(E-\beta_{2}E_{1}-E_{3}-F_{1}\right)\\ +(1-x)(1-z)\left(E-\beta_{2}E_{1}-E_{2}-E_{3}-F_{1}\right) \end{array}$$

$$U_{c}=yU_{c1}+(1-y)U_{c2}$$

Therefore, the dynamic equation for replication of the "active implementation" strategy adopted by the university is:

$$F(y)=\frac{dy}{dt}=y\left(U_{c1}-U_{c2}\right)$$

Similarly, let the expected rate of return of the village collective choosing the "efficient implementation" strategy be *U*_*v*1_, then the expected rate of return of the village collective choosing the "inefficient implementation" strategy be *U*_*v*2_, and the average expected return be *U*_*v*_, as follows:

$$U_{v1}=xy\left(G_{2}-V_{3}\right)+x(1-y)\left(-V_{2}-F_{2}\right)+(1-x)y\left(G_{2}-V_{1}-V_{3}\right)+(1-x)(1-y)\left(-V_{1}\right.\left.-V_{3}-F_{2}\right)$$

$$U_{v2}=x(1-y)\left(-V_{2}-F_{2}\right)+(1-x)y\left(-V_{1}\right)+(1-x)(1-y)\left(-V-V_{1}-V_{2}-F_{2}\right)$$

$$U_{v}=zU_{31}+(1-z)U_{v2}$$

Therefore, the replication dynamic equation for the village collective adopting the "efficient implementation" strategy is:

$$F(z)=\frac{dz}{dt}=z\left(U_{v1}-U_{v2}\right)$$

### 4.2 Stability analysis of the evolution of the strategies of the three main parties

For the government, universities and village collectives, there are E_1_ (0, 0, 0), E_2_ (0, 0, 1), E_3_ (0, 1, 0), E_4_ (0, 1, 1), E_5_ (1, 0, 0), E_6_ (1, 0, 1), E_7_ (1, 1, 0) and E_8_ (1, 1, 1) eight equilibrium points, but it is also necessary to discuss the asymptotic stability of these eight particular points. The Jacobi matrix can be used to analyze the stability of the differential equation [57, 58]. The specific Jacobi matrix is as follows:

$$\left[\begin{array}{ccc} (1-2x)\left[\alpha_{2}G_{1}-\alpha_{1}G_{1}-yG_{3}-zG_{2}\right] & -x(1-x)G_{3} & -x(1-x)G_{2}\\ y(1-y)G_{3} & (1-2y)\left(xG_{3}+zV_{3}-\beta_{2}E_{1}+\beta_{1}E_{1}\right) & y(1-y)V_{3}\\ z(1-z)(yV-V) & z(1-z)\left(xV-V-V_{3}+G_{2}\right) & (1-2z)\left(xyV-xV-y\left(V+V_{3}-G_{2}\right)+V\right) \end{array}\right]$$ (1)

The stability of each equilibrium point can be determined using the Lyapunov stability theory: The equilibrium point is asymptotically stable (ESS) if all eigenvalues of the Jacobi matrix are negative. If at least one of the eigenvalues of the Jacobi matrix is positive, the equilibrium point is unstable. If all the eigenvalues of the Jacobi matrix are negative except 0, the equilibrium is critical and the stability is uncertain, which cannot be determined from the sign of the eigenvalues [59, 60]. Substituting the above eight equilibrium points into the Jacobi matrix, the stability of each equilibrium point and its conditions are obtained (Table 3). When λ_1_ < 0, λ_2_ < 0 and λ_3_ < 0 of the equilibrium point are established at the same time, the equilibrium point can become the stable point of the system [61]. Therefore, only point E_3_ and point E_4_ may become the equilibrium point of the system.

**Table 3:** Stability judgment of each equilibrium point.

Balance point	λ_1_	λ_2_	λ_3_	Stability
*E*_1_(0,0,0)	v (+)	*α*_2_ *G*_1_-*α*_1_ *G*_1_(-)	*β*_1_ *E*_1_-*β*_2_ *E*_1_ (+)	Unstable
*E*_2_(0,0,1)	-v (-)	*V*_3_+β_1_ *E*_1_-*β*_2_ *E*_1_ (+)	*α*_2_ *G*_1_-*α*_1_ *G*_1_-*G*_2_ (-)	Unstable
*E*_3_(0,1,0)	*G*_2_-*V*_3_ (S)	*β*_2_ *E*_1_ -*β*_1_ *E*_1_ (-)	*α*_2_ *G*_1_-*α*_1_ *G*_1_-*G*_3_ (-)	satisfying (a) is ESS
*E*_4_(0,1,1)	*V*_3_-*G*_2_ (S)	*β*_2_ *E*_1_-*β*_1_ *E*_1_-*V*_3_ (-)	*α*_2_ *G*_1_-*α*_1_ *G*_1_-*G*_2_-*G*_3_ (-)	satisfying (b) is ESS
*E*_5_(1,0,0)	0	*α*_1_ *G*_1_-*α*_2_ *G*_1_(+)	*g*_3_+*β*_1_ *E*_1_-*β*_2_ *E*_1_ (+)	Unstable
*E*_6_(1,0,1)	0	*G*_2_+*α*_1_ *G*_1_-*α*_2_ *G*_1_ (+)	*G*_3_+*V*_3_+*β*_1_ *E*_1_-*β*_2_ *E*_1_ (+)	Unstable
*E*_7_(1,1,0)	*G*_2_-*V*_3_ (S)	*G*_3_+*α*_1_ *G*_1_-*α*_2_ *G*_1_ (+)	*β*_2_ *E*_1_-*β*_1_ *E*_1_-*G*_3_ (-)	Unstable
*E*_8_(1,1,1)	*V*_3_-*G*_2_ (+)	*β*_2_ *E*_1_-*V*_3_-*G*_3_-*β*_1_ *E*_1_ (-)	*G*_2_+*G*_3_+*α*_1_ *G*_1_-*α*_2_ *G*_1_ (+)	Unstable

Notes in the table denotes sign uncertainty; ESS denotes stable strategy; if condition (a) is not satisfied, then it is an unstable point. Condition (a): *G*_2_-*V*_3_ < 0. Condition (b): *V*_3_-*G*_2_ < 0.

It can be seen from Table 3. When satisfying *G*_2_-*V*_3_<0, the reward given by the government to the village collectives is less than the input of the village collectives, and E_3_ (0, 1, 0) is the stabilization point (ESS) of the replicated dynamic system. When *V*_3_-*G*_2_<0 is satisfied, E_4_ (0, 1, 1) is the ESS of the replicated dynamic system, at this time, the incentives given by the government to the village collectives should be higher than the inputs of the village collectives.

## 5 Evolutionary simulation analysis

### 5.1 Initial assignment of model parameters

**Fig 3:**
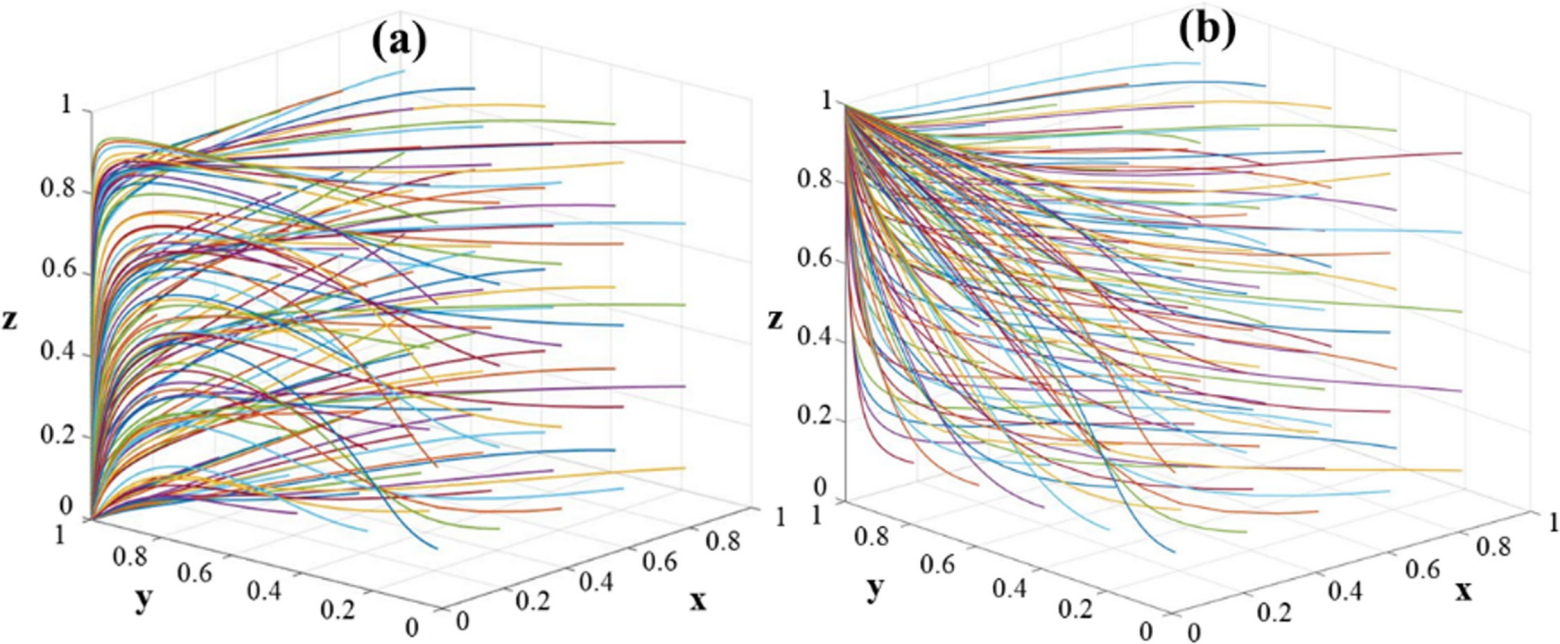
The evolutionary trajectory of the tripartite game model: (a) Evolution path of the stability point **E**_**3**_**(0,1,0)**; (b) Evolution path of the stability point **E**_**4**_**(0,1,1)**.5.2 Sensitivity analysis under policy changes.

The following numerical simulations were carried out using MATLAB 2018, which allows visualizing the dynamic evolution of the stakeholders in order to determine the best governance path for RRTT cultivation. In the stability analysis of the model, both E_3_ (0, 1, 0) and, E_4_ (0, 1, 1) may be the stability point of the model (Fig 3). The evolution process of the government, universities and village collectives is simulated to analyze the evolution process of each parameter. In this paper, the variables are assigned values so that G_1_ = 30, G_2_ = 3 (E_3_ stabilization point), G_2_ = 15 (E_4_ stabilization point), G_3_ = 5, E_1_ = 10, V_3_ = 8, V = 10, α_1_ = 0.8, α_2_ = 0.4, β_1_ = 0.8 and β_2_ = 0.4. The parameter setting is based on the research methods of Gao and Sun et al. [62, 63].

When satisfied *G*_2_-*V*_3_ < 0 (G_2_ = 3, V_3_ = 8), the simulation results are stabilized at (0, 1, 0), (weak dominance, active implementation, inefficient implementation) (Fig 3A). When satisfying *V*_3_-*G*_2_ < 0 (G_2_ = 15, V_3_ = 8), the simulation results are stabilized at (0, 1, 1), (weak dominance, active implementation, efficient execution) (Fig 3B). It shows that the stabilization results are independent of the initial strategies of the three parties. It shows that the simulation analysis is consistent and valid with the conclusions of the stability analysis of the strategies of the parties, which is a practical guidance for RRTT.

In this paper, we take the probabilities of the government, university and village collective choosing strong advantage, active implementation and efficient implementation as (x, y, z) = (0.5, 0.5, 0.5), respectively, and observe the changes of the government’s special financial input (G_1_), the incentive subsidy given by the government to the village collective (G_2_). government incentive subsidies given to the university (G_3_), and village collective input costs (V_3_) on the evolutionary trend of the game model. At the equilibrium points E_3_ (0, 1, 0) and E_4_ (0, 1, 0), the influence of government RRTT training special funds on the stability of the system is investigated.

**Fig 4:**
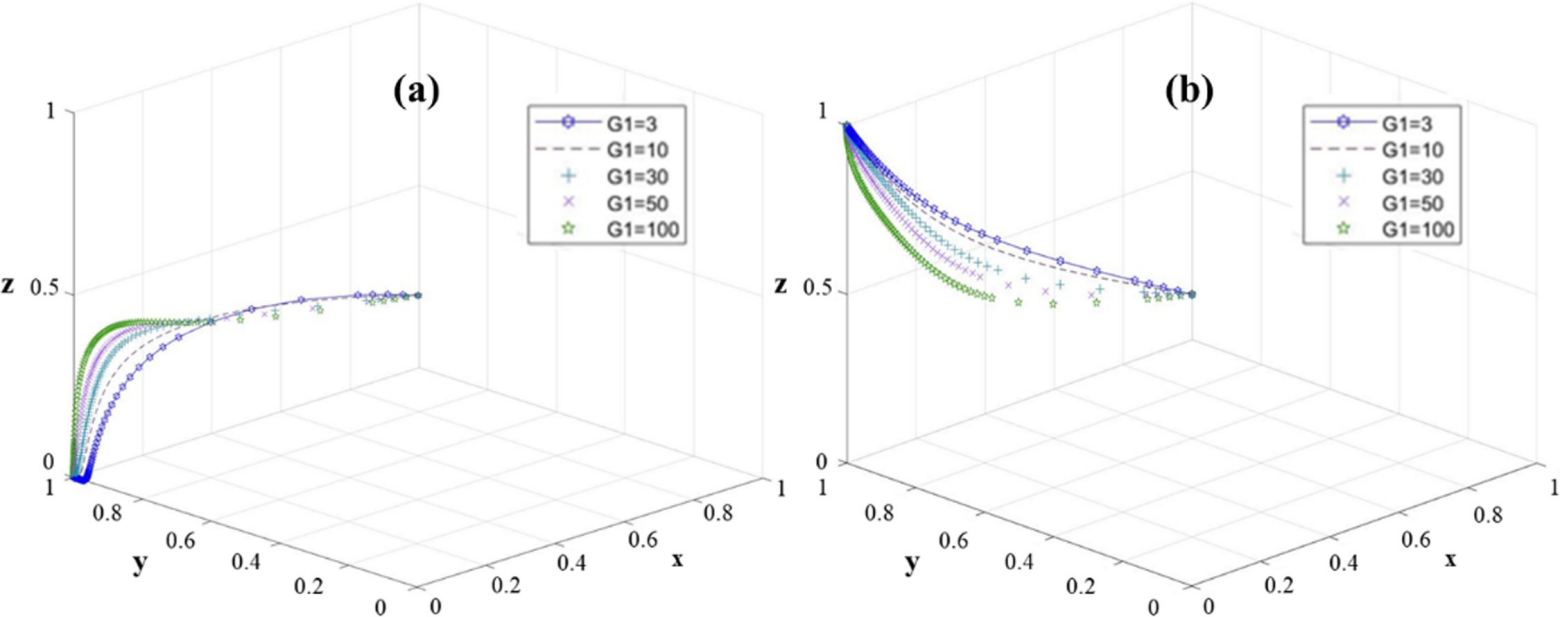
The sensitivity of the three-party game model to G_1_ change: (a) E_3_(0,1,0); (b) E_4_(0,1,1).

It can be seen from Fig 4A that with the increase of G_1_, the system will converge to the stable state E_3_ (0, 1, 0) more quickly. This means that a higher G_1_ value will prompt universities to implement projects more actively. Therefore, higher G_1_ is beneficial to the stability of the system. At this time, increasing the government’s financial investment in colleges and universities will only promote the active implementation of colleges and universities, but has little impact on the decision-making of village collectives. However, in the stable state E_4_ (0, 1, 1), regardless of the value of G_1_, the system eventually stabilizes at (0, 1, 1) (Fig 4B). It is worth noting that with the decrease of G_1_, village collectives are more likely to choose efficient execution strategies.

To sum up, the government needs to choose the appropriate financial investment strategy, while promoting the active implementation of the university, guide the village collective to choose efficient implementation, and avoid the situation that the government’s financial investment is disproportionate to the actual income.

In order to explore the influence of different reward strategies on the behavior decision-making of village collectives and universities, we designed four different parameter combinations, which represent the four models of ’low reward of village collectives-low incentive of universities’, ’low reward of village collectives-high incentive of universities’, ’high reward of village collectives-low incentive of universities’ and ’high reward of village collectives-high incentive of universities’.

**Fig 5:**
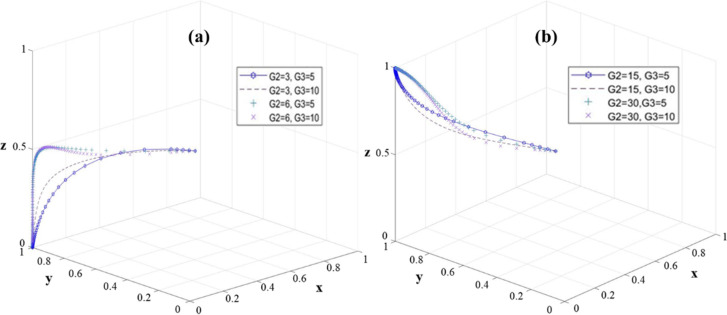
The sensitivity of the three-party game model to G_2_ and G_3_ change: (a) E_3_ (0,1,0); (b) E_4_ (0,1,1).

In the case of equilibrium points E_3_ (0, 1, 0) and E_4_ (0, 1, 0), we carried out simulation analysis, and the results are shown in Fig 5. At the E_3_ (0, 1, 0) equilibrium point (Fig 5A), when the government chooses to implement low incentives for village collectives (G_2_ = 3) and high rewards for universities (G_3_ = 10), although the probability of village collectives choosing to implement efficiently remains at 0, the probability of universities choosing to implement actively is stable at 1. This shows that the government’s implementation of low incentives for village collectives and high incentives for universities is difficult to effectively guide village collectives’ behavioral decision-making, but it can significantly improve the enthusiasm of universities. Therefore, this strategy can make the system reach the optimal decision state (0, 1, 0) faster. At the E_4_ (0, 1, 0) equilibrium point (Fig 5B), regardless of the government’s choice of reward strategy, the university and the village collective eventually choose the active implementation and efficient implementation mode, the probability is stable at 1, and the system reaches the optimal decision state (0, 1, 1). It is worth noting that under the low reward strategy for universities (G_3_ = 5), universities and village collectives achieve a steady state faster.

**Fig 6:**
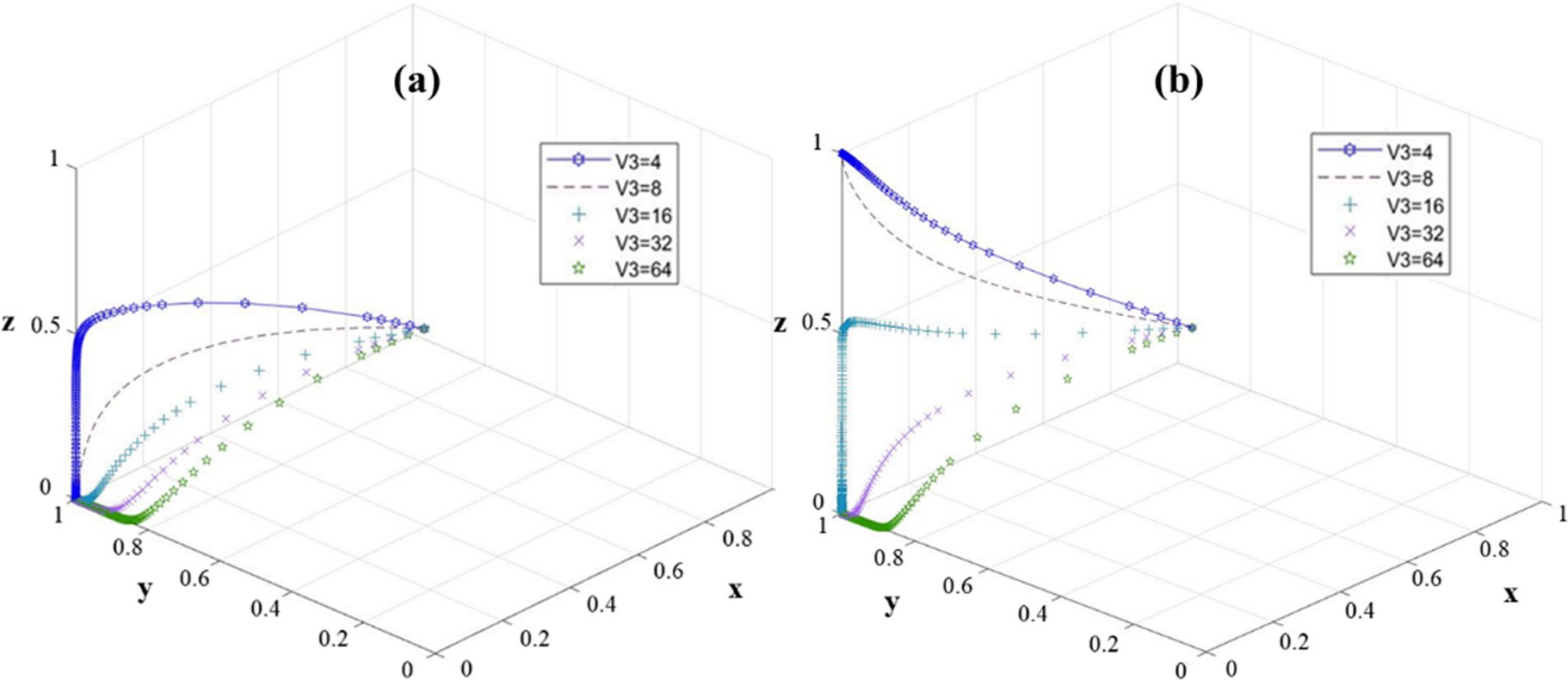
The sensitivity of the three-party game model to V_3_ change: (a) E_3_ (0,1,0); (b) E_4_ (0,1,1).

To sum up, the high reward strategy can effectively encourage universities to actively implement projects, but the incentive effect on village collectives is limited. Relatively speaking, improving the government’s incentive to village collectives (G_2_) and giving appropriate incentives to universities (G_3_) can more effectively encourage universities and village collectives to jointly choose an active and efficient implementation model, and the low reward strategy for universities is more conducive to the rapid stabilization of the system. In order to further explore the impact of changes in the village collective input cost (V_3_) on the stability of the system, we investigated the equilibrium points E_3_ (0, 1, 0) and E_4_ (0, 1, 0). The results are shown in Fig 6.

At the equilibrium point E_3_ (0, 1, 0) (Fig 6A), with the decrease of V_3_, the probability of the university stabilizes at 1 faster. When the input cost of the village collective is reduced, the university is forced to reach stability quickly. And with the increase of V_3_, the probability of village collective is stable at 0. Therefore, the change of V_3_ shows the opposite change rule to the change of university and village collective probability. At the equilibrium point E_4_ (0, 1, 1), with the change of V_3_, the system presents a certain degree of uncertainty, and the stable point gradually shifts during the evolution of the system (Fig 6B). It can be seen that when V_3_ is less than 8, the probability of village collective finally converges to 1, and with the decrease of V_3_, the faster the probability converges to 1. And when V_3_ is greater than 16, the probability of village collective eventually converges to 0. With the increase of V_3_, the probability converges faster to 0, and the system finally stabilizes. Therefore, when V_3_ is less than 8, the system can reach the ideal stable point E_4_ (0, 1, 1), indicating that the input cost of the village collective is the main factor affecting RRTT.

The decline of V_3_ mainly affects the behavior decision-making of village collectives and accelerates the evolution speed of village collectives’ choice of efficient execution decision-making. Therefore, the village collective should formulate a scientific and reasonable plan for the use of funds, reduce the cost of management and protection, and control it within 8, so as to improve the probability of active implementation of the village collective and accelerate the investment in rural revitalization and construction.

**Fig 7:**
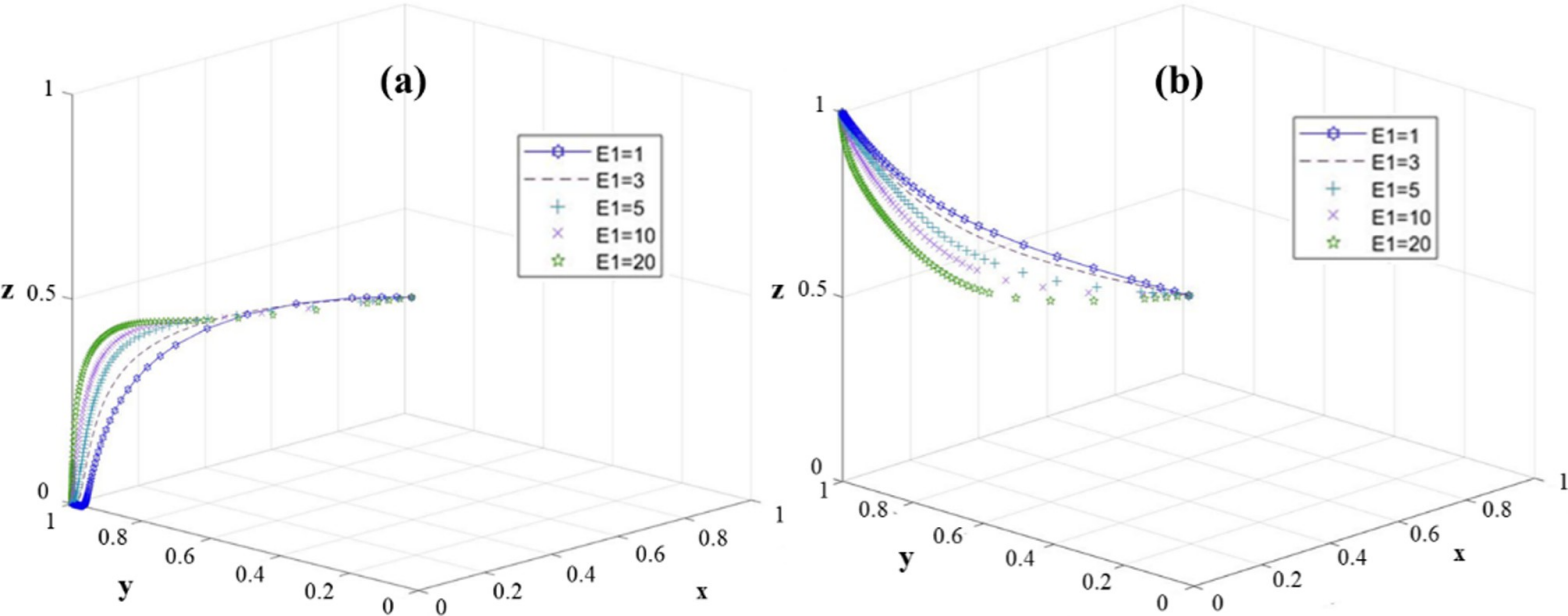
The sensitivity of the three-party game model to E_1_ change: E_3_(0,1,0); (b) *E*_4_(0,1,1).

In order to further explore the influence of the change of university input cost (E_1_) on the stability of the system, we investigate the equilibrium points E_3_ (0,1,0) and E_4_(0,1,0). The results are shown in Fig 7.

At the equilibrium point E_3_ (0, 1, 0), with the increase of E_1_, the system will be more quickly biased towards (0, 1, 0) (Fig 7A). Therefore, reducing E_1_ is conducive to the active implementation of colleges and universities. At this equilibrium point, the lower E_1_ is conducive to the stability of the system. At the equilibrium point E_4_ (0, 1, 0), no matter what value E_1_ takes, the system finally stabilizes at (0, 1, 1) (Fig 7B). However, with the decrease of E_1_, it is more conducive to the efficient implementation of university decision-making.

Therefore, at E_4_ (0, 1, 1), a lower E_1_ is beneficial to the stability of the system. At this point, the rise in government costs will not only make RRTT reach the ideal state, but will be counterproductive. This is because the high cost increases the government’s financial burden, but also reduces the possibility of choosing strong leadership, so that the village collective face the pressure of government assessment.

## 6 Discussion

Analysis of model setting. RRTT is most relevant to rural development. However, the main body of the game model design for rural revitalization is mostly concentrated in the government, rural areas and enterprises. For example, Fan [64] and Li [65] regard the government as one of the main participants. These studies attempt to promote rural development by encouraging and punishing other participants [23]. However, based on the complexity of the factors restricting RRTT and the current situation of information asymmetry among participants [66, 67], this paper takes China’s RRTT as the object, focuses on the regularity of RRTT development, and constructs an evolutionary game model. In addition, it is necessary to consider the game relationship related to talent training [35], so it is necessary to compare our research with other peer research results. Wang et al. [43] constructed a tripartite game model of government, university and enterprise. The scenario of this model is that the government subsidizes enterprises and universities, and provides policy support and fines to participants, which is similar to the scenario set in this study. However, the difference is that more studies lack the sensitivity parameter setting of key factors with policy changes in the training scenarios. This study distinguishes the dynamic changes of key parameters under different strategies of government and universities, and based on this, sets the government and universities to RRTT payment coefficient, which increases the possibility of practical application of the model.

Analysis of evolutionary strategy. In this study, according to the actual situation and related research distribution parameters [68], combined with the stability theorem of differential equations [69], the evolutionary strategy of each subject is analyzed by copying the dynamic model to obtain partial derivatives [70]. This evolutionary strategy makes all three parties become active participants, so that they can play a synergistic role in the training of rural revitalization talents. The above strategies provide a new perspective for us to understand the tripartite game process. This is different from previous studies that mostly focus on the separate role of government or universities in talent training, or simply consider village collectives as beneficiaries or passive participants [71].

About parameter sensitivity analysis. In this study, the sensitivity of the two stable points to the parameters is slightly different. At the (0, 1, 0) stable point, the government’s increase in investment only promotes the enthusiasm of the university, but has no effect on the positive role of the village collective. This is consistent with the research results that the government’s high investment is not conducive to the evolution of the system to the ideal state, and may even change its stable state [72]. However, it is worth noting that this stable point is not the best stable point of the system. The (0, 1, 1) stable point has higher operating efficiency and guiding role. When the government increases investment, it can simultaneously improve the enthusiasm of universities and village collectives and make the system more efficient. However, the government should consider the proportion of input and output in the process of high input, because blindly high-cost input will increase the financial burden of the government, reduce the enthusiasm of the government-led [73], and further increase the pressure on the village collective to face government assessment [74].

## 7 Conclusion and suggestion

### 7.1 Conclusion

RRTT is the fundamental premise and key foundation for realizing rural revitalization. Our research introduces a three-party evolutionary game model to analyze the behavioral interactions and stabilization strategies of the three stakeholders (government, universities and village collectives), and sensitivity of the main influencing factors. Three conclusions are obtained.

1. There is mutual influence between stakeholders, and the decision-making changes of each stakeholder will affect the decision-making of the other two stakeholders.
2. The stability point analysis shows that at (Weakly dominant, actively carry out, inefficient execution) strategy, only universities play an active role. Considering from the perspective of system stability and effectiveness, the model is an ideal equilibrium point at (Weakly dominant, actively carry out, efficient implementation). The model requires meeting the key condition that the input needs of village collectives are lower than the incentive subsidies given by the government.
3. From the sensitivity analysis results of key factors, the input cost of village collectives is the main factor affecting rural revitalization, and appropriate financial investment and incentive policies are more conducive to improving the probability of universities and village collectives.

### 7.2 Suggestion

According to the research results, the following suggestions are put forward for the cultivation of rural revitalization talents:

1. In view of the characteristics of mutual influence among stakeholders, the government should establish a long-term, perfect and supporting tripartite collaborative incentive mechanism, improve the talent supply and demand information platform between universities and village collectives, promote resource sharing and accurate docking of supply and demand, and provide platform support for the sustainable development of RRTT. The specific strategy is: the government first gives priority to improving the possibility of active implementation of village collectives through subsidies and policy support. At the same time, the government should further use preferential policy strategies to mobilize the enthusiasm of universities and rural collectives, and promote the cultivation of talents and the transformation of scientific and technological achievements in rural revitalization, so as to promote rural revitalization and the development of RRTT.
2. Combined with the conclusion of the stability point characteristic analysis, the government should ensure that the input cost of the village collective is lower than the incentive subsidy provided by the government. Therefore, the government should focus more on the input and construction of the village collective, and reduce the input cost of the village collective by developing the rural revitalization industry and reducing the cost of technology promotion, so as to promote the RRTT. At the same time, the government should also pay attention to mobilizing the development desire of the village collective and the demand for new technologies, and promote its demand for cooperation with universities. This close cooperation and collaborative innovation model of the government, universities and rural areas is expected to overcome the bottleneck of rural revitalization personnel training, realize the dynamic integration of talent training model and rural development, and provide strong talent support for rural revitalization.
3. From the sensitivity analysis of key factors, it can be seen that the government first chooses to increase the financial support for the village collective, and should appropriately increase the financial investment in the rural revitalization project, so as to improve the demand for rural technical talents and promote the village collective to increase the input cost of RRTT. And implement differentiated incentive policies, giving priority to giving more policy support to village collectives in the implementation of RRTT.

### 7.3 Limitations and prospects

Our research also has some limitations. The game evolution model is extremely complex, and the simplification and parameter setting of the model may affect the accuracy and applicability of the results. In addition, this study has not been deeply integrated with actual cases, and the explanatory power of the model and the effect of policy guidance still need to be further verified.

Our research focuses on the issue of rural revitalization talent training, which has important practical significance. This paper analyzes the particularity and complexity of rural talent training, and reveals the dynamic evolution law of rural talent training mechanism. In future research, the game model can be further refined, more realistic factors can be included, and the closeness of the model can be improved. Comparative research can also be carried out to analyze the similarities and differences of rural talent training in different types of areas, and put forward targeted optimization strategies. Enrich the talent training ideas for rural revitalization and development.
